# Contained Morcellation: Review of Current Methods and Future Directions

**DOI:** 10.3389/fsurg.2017.00015

**Published:** 2017-03-14

**Authors:** Enes Taylan, Cagdas Sahin, Burak Zeybek, Ali Akdemir

**Affiliations:** ^1^Laboratory of Molecular Reproduction and Fertility Preservation, Division of Reproductive Medicine, Department of Obstetrics and Gynecology, New York Medical College, New York, NY, USA; ^2^Department of Obstetrics and Gynecology, Ege University School of Medicine, Izmir, Turkey; ^3^Department of Obstetrics and Gynecology, University of Texas Medical Branch, Galveston, TX, USA

**Keywords:** contained morcellation, enclosed morcellation, in-bag morcellation, laparoscopy, hysterectomy, myomectomy

## Abstract

Power morcellation of surgical specimen during laparoscopic surgery is a practical technology that provides the opportunity to perform several minimally invasive procedures. However, this technology brought forward additional risks and complications associated with dissemination of both benign and malignant tissues inside the abdominal cavity. Based on startling cases, Food and Drug Administration (FDA) announced a discouraging statement on the use of power morcellators that decreased the number of minimally invasive approaches in the following period. As a response to these concerns and negative impacts of the FDA statement, researchers developed several new approaches resulting in contained or in-bag morcellation methods. In this review, we aimed to discuss these current methods and provide an insight for future developments.

## Introduction

Minimally invasive surgery (MIS) has dramatically changed the vision of modern surgical approach. Advantages of MIS in the treatment of a diverse range of gynecological conditions have been well demonstrated by several studies in the last few decades. These well-documented advantages include less perioperative complications, better cosmetic results, faster recovery, and improved quality of life compared to laparotomy (1). In the United States, according to the recent reports, almost half of the annual hysterectomy procedures for benign gynecologic indications were performed *via* MIS (2). In addition, thousands of women underwent myomectomy and benefited from the virtues of advanced minimally invasive methods in the last few decades.

Minimally invasive surgery techniques involve vaginal, laparoscopic (conventional multiport or single site), and robotic surgical approaches. Their advantages over open surgeries in the treatment of a wide range of gynecologic diseases have been acknowledged. As a consequence, American College of Obstetricians and Gynecologists and American Association of Gynecologic Laparoscopists recommended the use of vaginal or laparoscopic approaches in hysterectomy for the treatment of benign gynecologic diseases (3, 4).

Along with the significant technological advances and expanded application fields, MIS brought an inherent challenge, the safe extraction of surgical specimen, which is also the primary focus of this review. Developed surgical techniques improved our capabilities and also brought serious risks and legal litigations. In the following sections, we have discussed our current knowledge of morcellation methods, their potential risks, and promising solutions as future directions.

## Tissue Morcellation and Potential Risks

From past to the present, MIS techniques rapidly advanced along with innovative solutions developed when surgeons confronted by obstacles. At the time of the uterine specimen (uterine fibroid or uterus itself) removal from the abdominal cavity, the surgical specimen is frequently too large to be extracted through the routes used to access the surgical field without impairing the tissue integrity. To accomplish the operation overcoming this challenge, an additional process named morcellation has been described. Morcellation procedure can be defined as fragmentation of the large tissue specimen into smaller pieces to facilitate the specimen extraction (5). This procedure can be performed either manually using a surgical scalpel or by using a specifically designed electromechanical device.

Power morcellation (PM), also known as electromechanical morcellation, has been introduced to the modern surgical practice in 1993 and utilized in various specialties including gynecology, general surgery, and urology after the approval of the US Food and Drug Administration (FDA) in 1995 (6, 7). Power morcellators are specifically designed to transform electrical energy into mechanical power, which is needed for cutting the surgical specimen into smaller pieces to be easily removed through a 12–20 mm laparoscopic access port. Since its first application, various types of power morcellator with different features in design, weight, working principle, blade size, and cutting speed have been developed (8). Currently, there are 11 different commercially available devices some of which have been reported to have higher rates of morcellation performance (7). However, there is lack of evidence demonstrating that any one of the devices is associated with higher risks of organ injuries or tissue dissemination.

Severe complications mostly involved bowels and vascular structures caused by the spinning blade of the morcellator were reported (9). Also, performing intracorporeal PM can lead scattering of benign tissues such as leiomyoma and endometriosis. Dispersed tissue fragments may implant on abdominal organ surfaces leading to inflammation, infection, and intestinal obstruction, which require additional surgical interventions and treatments (10–12). However, among the concerns the most formidable one that brought this technology under scrutiny is unintentional dissemination of malignant cells, which can lead severe consequences such as worsening the prognosis by upstaging the occult cancer. Recently, iatrogenic dissemination of unexpected malignancies such as sarcomas and adenocarcinomas after intracorporeal morcellation has been reported and draw the attention of popular media around this surgical method (13–15). After two decades of surgical practice with power morcellators, FDA released a warning statement discouraging the use of PM in women undergoing hysterectomy and myomectomy based on the safety concerns (16).

Uterine cancers are the most common gynecologic malignancies in the United States with an estimation of more than 10,000 deaths in 2016 (17). Endometrial cancers are usually diagnosed in women at older ages and are uncommon in reproductive ages. They are typically identified by abnormal bleeding and can be diagnosed with an appropriate preoperative evaluation using imaging and endometrial sampling. However, uterine sarcomas are very rare with an incidence of 7–8% of all uterine cancers and there is no currently available method for an accurate preoperative diagnosis (18). Although the occurrence of uterine sarcomas is very rare in women younger than 40 years, its risk factors are not well understood. They are usually discovered postoperatively and, regardless of the tumor stage, the prognosis is very poor with only 40–66% survival at 5 years (19, 20).

The risk of unanticipated uterine sarcoma in patients undergoing a uterine morcellation was 0.22% in a retrospective cohort study (21). Recently, Wright et al. investigated the prevalence of underlying uterine malignancies in women who underwent myomectomy in a large nationwide retrospective study, which included more than 40,000 patients from 496 hospitals. The prevalence of uterine cancer in women who underwent myomectomy with and without PM during surgery was 0.09 and 0.18%, respectively (22). Although the overall risk of occult malignancy appears to be very low, PM should be used with caution in older patients, as the risk significantly increases with age.

In patients with suspicion for occult uterine malignancy after preoperative evaluation, alternatives to morcellation should be recommended. In these cases, minilaparotomy or vaginal extirpation of the uterine specimen is more reliable option for tissue extraction as the patient safety is the main priority. Moreover, it should be emphasized that there is no currently available method for tissue extraction that completely eliminates the risk of cellular dissemination.

## Contained Tissue Extraction

In an Italian survey, Mandato et al. reported that 58.7% of gynecologists declared that they would change their surgical practice after FDA safety communication only to prevent legal litigation (23). In another recent study from US, comparing the number of cases performed during 8 months before and after FDA warning statement, authors reported 5.8 and 19% decrease in the number of minimally invasive hysterectomy and myomectomy procedures, respectively (24). More concerning, Harris et al. reported that the number of major surgical complications and hospital readmissions were significantly increased with the decreasing numbers of minimally invasive hysterectomy. These results, from a statewide surgical cohort study, can be translated to an additional $23 million burden to annual health care costs (25). In response to these negative impacts on surgical outcomes and health care costs, surgeons embarked on a quest to overcome the challenges and eliminate tissue dissemination during morcellation (26–44).

### Abdominal Approach to Contained Tissue Extraction

In 2014, Einarsson et al. described “Sydney in bag morcellation technique” as a contained abdominal morcellation method for multiport laparoscopic surgery (26). In this technique, using two different tissue retrieval systems—a 15-mm EndoCatch bag (Covidien, Mansfield, MA, USA) and Anchor TRS-200 tissue retrieval system (Anchor Surgical, Addison, IL, USA)—authors placed the surgical specimen inside the retrieval bag and inserted a 12-mm trocar into the bag through the umbilicus followed by insufflation. Subsequently, a 5-mm balloon-tip Kii trocar (Applied Medical, Rancho Santa Margarita, CA, USA) pierced into the insufflated bag with the specimen inside. Power morcellator was introduced through the umbilicus incision and morcellation performed under optic visualization. Once morcellation has been completed, the isolation bag was removed through the umbilical port after deflation of trocar balloon.

The Sydney in-bag morcellation technique presents a novel approach for contained PM. However, it poses a potential risk of tissue dissemination arising from the piercing of the isolation bag. Penetrating the bag inside the abdominal cavity jeopardizes the bag integrity and may result in tissue leakage. Therefore, we developed an innovative method for enclosed morcellation using a surgical glove (27). Inspired by the topological shape of the surgical glove, we aimed to create an isolated space with protrusions that can be manipulated through the ports on abdominal wall. In our technique, fingertips of the surgical glove were exteriorized through the trocars that enabled direct access to the isolated space without disrupting the surface integrity of the containment. However, our technique was limited with the volume and elasticity of the surgical glove, as it is not specifically designed for this purpose. Inspiring from this method, Rimbach et al. developed a novel isolation bag (More-Cell-Safe, A.M.I., Austria) with two openings and successfully applied it in laparoscopic PM of the uterus. The larger opening was used for placement of the uterine tissue inside the bag and introduction of the power morcellator as well, while the smaller opening was used to insert the laparoscope (28). Recently, Paul et al. described the use of a specially designed isolation bag using a similar bag shape (MorSafe; Veol Technologies, Mumbai, India) for two-port morcellation method (29).

Morcellation of uterine specimen within an insufflated isolation bag during single-site laparoscopic surgery was also recently described. Using a cordless electric morcellator, the LiNA Xcise (LiNA Medical, Glostrup, Denmark), introduced through a 5-mm trocar, uterine specimens of 12 patients were morcellated in a contained fashion without any complications (30). As it requires neither bag penetration nor piercing with trocar, this technique appears as a reliable approach.

Contained PM of uterine specimen in multiport laparoscopy was associated with 20–26 min longer operative time when compared with those without PM (31, 32). However, no significant differences related to estimated blood loss, length of hospital stay, and perioperative complications were recorded. On the other hand, Venturella et al. reported operative times similar to in-bag manual morcellation without PM (33). These reported variations in operative times are possibly due to the differences in morcellation technique, tissue size, and surgical experience. The current body of evidence suggests that contained PM is a time efficient and feasible method in laparoscopic surgery.

To evaluate the safety of contained PM in terms of tissue dissemination, Cohen et al. conducted both *in vivo* and *in vitro* studies (34, 35). Although investigators detected dye leakages in a few trials, PM in an isolation bag was suggested as a feasible method with the need of further studies to confirm the safety of current techniques and materials.

### Vaginal Approach to Contained Tissue Extraction

Vaginal channel is a natural orifice through which an abdominal specimen can be easily removed after creation of colpotomy or culdotomy incision. Vaginal route can provide removal of entire uterus without morcellation especially in oncological cases. However, in cases with severe vaginal atrophy, narrow pelvic arch, nulliparity, and bulky uterus, the tissue removal can be challenging. When uterine specimen is too large to be extracted, intact morcellation can be performed through vagina. Vaginal morcellation is relatively faster and simple to learn and perform. Commonly used vaginal morcellation techniques include bivalving, wedge resection, coring, myomectomy, and recently described paper roll method (36, 37). Moreover, vaginal morcellation can be performed within a containment bag to prevent tissue dissemination. After either abdominal or vaginal insertion of containment bag in various types, authors accomplished vaginal morcellation in an enclosed fashion (38–41). Also, vaginal retractors can be used to facilitate extraction providing better exposure.

Besides its efficiency, vaginal tissue extraction should be performed in experienced hands as it carries the risks of bladder, rectum, and vaginal lacerations (38). Therefore, meticulous inspection of the surgical field should be done to exclude possible injuries after removal of the specimen. Although contained vaginal morcellation may prevent the risk of tissue dissemination, Solima et al. reported that in 4 of 12 cases, the containment bags were found to be ruptured after filling up with methylene dye (42). Demonstrating potential risk of tissue dissemination even in contained vaginal morcellation, authors addressed the importance of development of new, resistant, and durable materials and devices.

## Summary and Future Directions

Morcellation of the large surgical specimen is a crucial step in the accomplishment of minimally invasive procedures and utilization of power morcellator facilitates this frequent time-consuming process. However, it should be noted that performing uncontained intracorporeal morcellation of uterine specimen either *via* vaginal or abdominal approach has been associated with tissue dissemination (43). After PM in laparoscopic myomectomy, Toubia et al. detected spindle cells in post-morcellation peritoneal washing samples (44). Therefore, morcellation of the specimen in an enclosed fashion should be the preferred method as many developed techniques and several specimen retrieval bags for this method have been widely available. Nonetheless, in patients with suspicion for occult uterine malignancy after an appropriate preoperative evaluation, morcellation should be avoided (45). In those cases, alternatives such as minilaparotomy or vaginal retrieval of the uterine specimen are more reliable options.

Vaginal extraction of surgical specimen in a containment bag is a feasible option, besides it still carries the risk of adjacent pelvic organ injuries and bag rupture. Although it has not been described yet, vaginal PM of uterine specimen can be a hypothetical solution to overcome these limitations. We believe, by modifying our previously described method, this hypothetical approach can be actualized in experienced hands. However, larger bags made with resistant and elastic materials are needed to realize this method.

As the main concern with morcellation is the spillage of malignant cells, it should be realized that cellular dissemination is most likely initialized by the surgical procedure itself. Spindle cells after myomectomy have been detected in peritoneal washings even in the absence of the morcellation (27, 35, 44). Although its clinical significance is still unclear, patients should be informed that there is an inherent risk of cellular dissemination during myomectomy procedure regardless of morcellation. In this point, we believe that an advanced innovative surgical method providing an enclosed space not only for the morcellation procedure but also for the preceding myomectomy procedure can be developed in the future. This futuristic view may provide an insight for the future developments of new surgical methods and devices.

In conclusion, it should be emphasized that there is currently no available method for tissue extraction that completely eliminates the risk of cellular dissemination. Therefore, further investigations and technological developments are needed to improve morcellation technique. Collaboration between surgeons, device manufacturers, and designers should also be encouraged to find innovative solutions.

## Author Contributions

All authors listed have made substantial, direct, and intellectual contribution to the work and approved it for publication.

## Conflict of Interest Statement

The authors declare that the research was conducted in the absence of any commercial or financial relationships that could be construed as a potential conflict of interest.
